# Serum fetuin‐A and Ser312 phosphorylated fetuin‐A responses and markers of insulin sensitivity after a single bout of moderate intensity exercise

**DOI:** 10.14814/phy2.14773

**Published:** 2021-03-02

**Authors:** Guang Ren, Robert L. Bowers, Teayoun Kim, Alonzo J. Mahurin, Peter W. Grandjean, Suresh T. Mathews

**Affiliations:** ^1^ Department of Nutrition and Dietetics Auburn University Auburn AL USA; ^2^ School of Kinesiology Auburn University Auburn AL USA; ^3^ Department of Nutrition and Dietetics Samford University Birmingham AL USA

**Keywords:** fetuin‐A, insulin sensitivity, obesity, phosphofetuin‐A, single bout exercise

## Abstract

Fetuin‐A (Fet‐A), secreted by the liver and adipose tissue, inhibits insulin receptor tyrosine kinase activity and modulates insulin action. Numerous studies have shown association of elevated serum Fet‐A concentrations with obesity, non‐alcoholic fatty liver disease, and type 2 diabetes. Both moderate body weight loss (5%–10%) and significant body weight loss have been shown to decrease serum Fet‐A and improve insulin sensitivity. Currently, there are no studies examining the effects of a single bout of exercise on serum Fet‐A or Ser312‐pFet‐A (pFet‐A) responses. We hypothesized that a single bout of moderate‐intensity exercise will lower serum Fet‐A and that these changes will be associated with an improvement in insulin sensitivity. Thirty‐one individuals with obesity and 11 individuals with normal body weight were recruited. Participants underwent a single bout of treadmill walking, expending 500 kcal at 60%–70% VO_2max_. Oral glucose tolerance tests (OGTT) were administered before the single bout of exercise (Pre Ex) and 24 h after exercise (24h Post Ex). In individuals with obesity, we observed a transient elevation of serum Fet‐A concentrations, but not pFet‐A, immediately after exercise (Post Ex). Further, a single bout of exercise decreased glucose_AUC_, insulin_AUC_, and insulin resistance index in individuals with obesity. Consistent with this improvement in insulin sensitivity, we observed that Fet‐A_AUC_, pFet‐A_AUC_, 2 h pFet‐A, and 2 h pFet‐A/Fet‐A were significantly lower following a single bout of exercise. Further, reductions in serum Fet‐A_AUC_ 24h Post Ex were correlated with a reduction in insulin resistance index. Together, this suggests that alterations in serum Fet‐A following a single bout of moderate‐intensity endurance exercise may play a role in the improvement of insulin sensitivity.

**Clinical Trial Registration:**

NCT03478046; https://clinicaltrials.gov/ct2/show/NCT03478046.

## Graphical abstract

Serum Fet‐A was transiently elevated immediately after a single bout of exercise. and restored to Pre Ex levels 24 h after a single bout of exercise. Furthermore, serum Fet‐A_AUC_, pFet‐A_AUC_, glucose_AUC_, insulin_AUC_, and insulin resistance index were significantly decreased 24 h after a single bout of exercise compared with Pre Ex levels in individuals with obesity. Additionally, the reduction in 24 h Post Ex insulin resistance index was correlated with a reduction in serum Fet‐A_AUC_, suggesting that alterations in serum Fet‐A and its phosphorylated form, Ser‐312 pFet‐A, may potentially contribute to the observed improvement in insulin sensitivity following a single bout of endurance exercise.

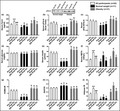

## 1 INTRODUCTION

The benefits of regular physical activity and maintenance of a healthy weight in glycemic control and prevention of obesity and type 2 diabetes have been well documented (Helmrich et al., 1994; Qiu et al., 2012; Schulze & Hu, 2005). It has been well established that a single bout of endurance exercise effectively stimulates whole body insulin sensitivity and glucose tolerance, and that this effect can persist from 2 to 48‐h post exercise (Devlin et al., 1987; Devlin & Horton, 1985; Mikines et al., 1988a; Perseghin et al., 1996). Several metabolic and hemodynamic factors can contribute to improvements in glucose homeostasis observed after an acute exercise in insulin‐resistant individuals, including enhanced insulin action on skeletal muscle glucose uptake, GLUT4 translocation, GLUT4 mRNA expression, reduced hepatic glucose production, and improved blood flow to skeletal muscle (Egan & Zierath, 2013; Henriksen, 2002; Kraniou et al., 2000; Thorell et al., 1999).

Organokines, secreted by specific tissues, with both paracrine and endocrine functions have been shown to regulate different signaling pathways and may play important roles in development of insulin resistance and type 2 diabetes (Hotamisligil, 2006). Exercise has been shown to elicit beneficial adaptive responses in many organs that play an important role against chronic conditions such as obesity (Booth et al., 2012). Interleukin (IL)‐6 has been identified as the predominant myokine whose concentrations transiently increase up to 100‐fold after prolonged exercise, and only modestly after shorter duration exercise (Fischer, 2006; Pedersen & Febbraio, 2008). However, the relationship between exercise and hepatokines is limited (Stefan & Haring, 2013). A recent study has shown that moderate intensity exercise increased plasma concentrations of hepatokines FGF‐21 and follistatin, 4 h and 7 h post exercise, respectively (Willis et al., 2019).

Human fetuin‐A (Fet‐A), also known as alpha2‐HS glycoprotein (AHSG), a hepatokine, primarily released from the liver and present in the blood of adults at concentrations ranging from 300 to 600 μg/ml (Dziegielewska et al., 1990; Lebreton et al., 1979; Putman, 1984), was originally identified as a physiological inhibitor of insulin receptor tyrosine kinase (IR‐TK) and IR‐autophosphorylation in skeletal muscle and liver tissue (Auberger et al., 1989; Goustin & Abou‐Samra, 2011; Goustin et al., 2013; Mathews et al., 1997, 2000, 2006; Srinivas et al., 1993). Recently, Fet‐A was identified as an endogenous ligand of Toll‐like receptor 4 (TLR4) that stimulates inflammation (Pal et al., 2012), and was described as a key hepatokine that regulates insulin action and inflammation (Stefan & Haring, 2013). Elevated circulating Fet‐A levels were shown to be associated with obesity, insulin resistance, and an increased risk for type 2 diabetes (Ishibashi et al., 2010; Ix et al., 2006, 2008; Mori et al., 2006; Stefan et al., 2008; Weikert et al., 2008).

Previously, it was shown that adults who were habitually sedentary had higher plasma Fet‐A compared with physically active counterparts (Jenkins et al., 2011). Recent studies by Malin et al., (2014) have suggested that short‐term exercise training, without body weight changes, significantly decreased circulating fetuin‐A, which was correlated with the improvement of insulin‐regulated glucose uptake. Another recent study by the same authors indicate that 12 weeks of exercise training lowered fetuin‐A by 8%, and this was correlated with lower hepatic insulin resistance, increased metabolic flexibility, and HMW‐adiponectin.

Human circulating Fet‐A exists in both phosphorylated (approximately 20%) and dephosphorylated (approximately 80%) forms (Dziegielewska et al., 1990; Haglund et al., 2001). Fetuin‐A is phosphorylated on Ser120 and Ser312, with the majority of phosphorylation on Ser312 (~77%) (Haglund et al., 2001). Recently, we have shown that phosphorylation status of Fet‐A (pFet‐A) was critical for its inhibitory effects on insulin action and that elevated pFet‐A concentrations were correlated with obesity and insulin resistance (Ren et al., 2019). Furthermore, a moderate 8% –10% body weight loss significantly decreased serum pFet‐A, pFet‐A:Fet‐A ratio, and was associated with an improvement of insulin sensitivity (Ren et al., 2020).

Prior studies have not examined the effects of a single bout of exercise on serum pFet‐A and none of them have characterized serum pFet‐A or Fet‐A immediately post exercise to 24 h after exercise to understand the timecourse effects of exercise. Therefore, the goal of this study was to determine alterations in serum Fet‐A and pFet‐A post exercise, and 24 h post exercise in normal weight and in individuals with obesity and correlate these changes with surrogate markers of insulin sensitivity. We hypothesized that a single bout of exercise would lead to alterations in serum Fet‐A and pFet‐A concentration and that these changes would be associated with the improvement of surrogate markers of insulin sensitivity.

## 2 METHODS

### 2.1 Study population

A total of 31 individuals with obesity and 11 normal‐weight individuals were recruited for this study, as reported previously (Ren et al., 2019). All volunteers met the following criteria: between 30 and 65 years of age, non‐smokers, no reported cardiovascular or metabolic disease, not currently taking medication known to alter lipid or glucose metabolism, weight stable for the past 6 months, and did not engage in regular physical activity for the past 6 months. Informed consent was obtained from all subjects, and this study received approval from the Auburn University Institutional Review Board (07‐210 MR 0710) and was registered on ClinicalTrials.gov (NCT03478046).

**FIGURE 1:**
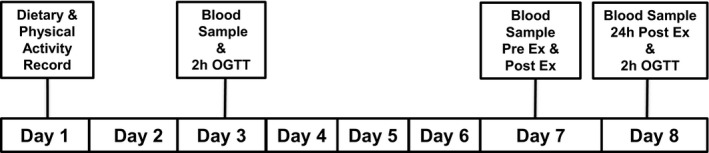
Timeline for blood sampling and single bout of exercise. On day 3, an oral glucose tolerance test was administered. On day 7, participants completed a single bout of endurance exercise expending 500 kcal, 60%–70% VO_2max_. Fasting blood samples were obtained pre‐exercise (Pre Ex), post exercise (Post Ex), and 24 h after the single bout of exercise (24h Post Ex) to assess Fet‐A, pFet‐A, and markers of insulin sensitivity. An oral glucose tolerance test was administered 24h Post Ex to analyze area under the curve (AUC) for glucose, insulin, insulin resistance index, Fet‐A, and pFet‐A

After the preliminary screening procedures were carried out on the first day, subjects reported to the laboratory on the 3^rd^ day in the fasted state (12‐h fast restricted to water only) (Figure 1). Participants were asked to record their diet and physical activity for 3 days. These records were analyzed using the Food Processor software (Esha Research). Participants were counseled to maintain a similar energy and nutrient intake and to refrain from any kind of exercise during the blood sampling timeline. Participants were counseled to maintain a similar energy and nutrient intake and to refrain from any kind of exercise during the blood sampling timeline (Figure 1). A standard oral glucose tolerance test (OGTT) was administered with 75 g glucose. Blood samples were drawn at zero time and 30 min intervals for 2 h following consumption of 75 g glucose to measure blood glucose, serum insulin, serum Fet‐A, and pFet‐A. All blood samples were obtained by venipuncture from an antecubital vein. Aliquots of serum were stored in −80°C freezer until further analyses.

### 2.2 Single bout of exercise

On day 7 (Figure 1), participants returned to the laboratory after a 12‐h fast (restricted to water only) for pre‐exercise blood sampling (Pre Ex). Participants walked or jogged on a motorized treadmill (Trackmaster TMX425, Newton, KS) at 60%–70% of their VO_2max_ for the duration required to expend 500 kcals of energy. Blood samples were taken immediately after exercise (Post Ex). To examine the sustained effects of the single bout of exercise, all subjects reported to the laboratory the next day, i.e., 24 h following the single bout of exercise (24 h Post Ex), i.e., day 8, and a fasting blood sample was collected. Immediately after the fasting blood sample collection, all participants were administered a second OGTT following the same protocol as described previously.

### 2.3 Serum fetuin‐A and Ser312‐phosphorylated fetuin‐A

Serum Fet‐A concentrations were assayed in duplicate using an ELISA kit (BioVendor, LLC). Human fetuin‐A standards ranging from 2 to 100 ng/ml, quality controls, and 1:10,000 diluted serum samples were added to anti‐human fetuin‐A antibody‐coated microtiter strips. Absorbance was read in a microplate reader at 450 nm. As a human pFet‐A ELISA was not commercially available, we assayed this by Western blotting. Serum samples diluted 1:100 in sterile saline and proteins were separated on a 4%–20% gradient SDS–PAGE gel (Bio‐Rad), transferred to nitrocellulose membrane, blocked in 5% non‐fat dry milk (Bio‐Rad), and serum pFet‐A was detected using a custom‐generated affinity‐purified antibody (Affinity BioReagents) against the phosphorylated Ser312‐fetuin‐A epitope “HTFMGVVSLGSPS(PO4)GEVSHPR.” Chemiluminescence was imaged with UVP BioImaging system (UVP), and area densities of the bands were analyzed using UnScan‐It software package (Silk Scientific). To assure a standardized quantitation of data and to account for variation in gel‐to‐gel exposures/density quantitation, serum pFet‐A band densities from samples were compared to band pixels of a “normal body weight quality control” serum sample, which was loaded in duplicate on every gel. This approach offered an objective and comparable quantitation of serum levels of pFet‐A. Statistical analyses were performed using fold change data compared agaisnt “normal body weight quality control” serum sample.

### 2.4 Biochemical analysis, insulin resistance, and glucose tolerance

Serum glucose (Glucose hexokinase assay kit, Cliniqa Corporation), serum insulin (Millipore Corporation), serum non‐esterified fatty acids (NEFA assay kit) were assayed as reported earlier (Ren et al., 2020). Homeostasis model assessment of insulin resistance (HOMA‐IR), a reflection of hepatic insulin resistance, was calculated using the following formula: [fasting insulin (μU/ml × fasting glucose (mmol/L)/22.5] (Matthews et al., 1985); adipose insulin resistance (Adipose‐IR) was calculated as follows: [fasting NEFA mEq/L × fasting insulin µU/ml] (Abdul‐Ghani et al., 2008); quantitative insulin sensitivity check index (QUICKI), a measure of insulin sensitivity, was calculated by [1/(log (fasting insulin µU/ml) + log (fasting glucose mg/dl))] (Katz et al., 2000); glucose to insulin ratio (GIR) was calculated as glucose (mg/dl)/insulin (µU/ml). Total area under the curve for glucose (glucose_AUC_) and insulin (insulin_AUC_) were calculated using the trapezoidal method, by calculating the sum of areas of all equivalent rectangles. Insulin resistance index, which primarily reflects skeletal muscle glucose uptake, was estimated by multiplying insulin_AUC_ and glucose_AUC_ and dividing by 10^6^ (Evans et al., 2001).

### 2.5 Statistical analysis

An unpaired Student's *t* test was used to determine statistical differences between baseline characteristics in individuals with normal‐weight and individuals with obesity. A two‐way ANOVA was used to determine statistically significant differences within intervention groups Pre Ex, Post Ex, 24 h Post Ex and between normal weight and obesity groups. We used the Bonferroni multiple comparison test to identify significant differences. A paired Student's *t* test was used to identify statistical differences in AUCs between Pre Ex OGTT and 24  h Post Ex OGTT for all participants (both individuals with normal weight and individuals with obesity). Pearson product moment correlation coefficients were used to examine associations, and significance was accepted as *p* < 0.05. Data are expressed as mean ± SD, unless noted otherwise.

## 3 RESULTS

### 3.1 Effect of a single bout of exercise on serum Fet‐A, pFet‐A, pFet‐A/Fet‐A, and surrogate markers of insulin sensitivity

**FIGURE 2:**
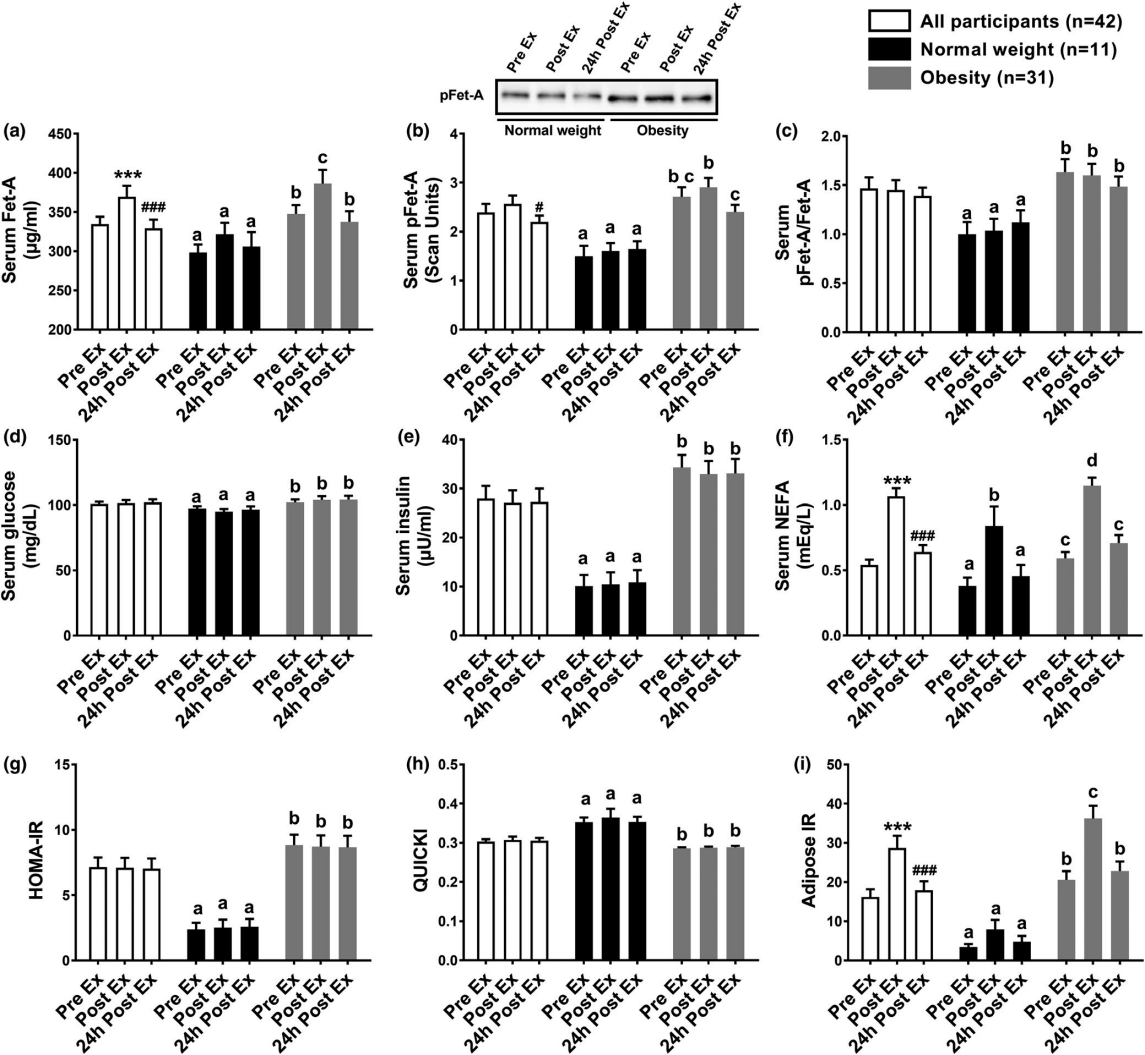
Fasting serum Fet‐A, pFet‐A, pFet‐A/Fet‐A, and metabolic indices individuals with normal weight (*n* = 11) and individuals with obesity (*n* = 31) and all participants (*n* = 42) before exercise (Pre Ex), immediately after exercise (Post Ex), and 24 h after a single bout of exercise expending 500 kcal (24h Post Ex). A representative Western blot depicting alterations in serum pFet‐A, from Pre Ex, Post Ex, and 24h Post Ex timepoints in an individual with normal weight and an individual with obesity is shown (inset). NEFA: non‐esterified fatty acids; HOMA‐IR: Homeostasis model assessment of insulin resistance; Adipose‐IR: adipose insulin resistance; and QUICKI: Quantitative insulin sensitivity check index. Data are expressed as Mean ± SEM. Statistical significance for comparisons of all participants are shown as follows: ^***^Pre‐Ex vs Post Ex, *p* < 0.001; ^###^Post Ex vs 24h Post Ex, *p* < 0.001; ^#^Post Ex vs 24 h Post Ex, *p* < 0.05. In individuals with normal weight or obesity, different letters in superscript following values indicate statistical significance, *p* < 0.05

We previously reported baseline anthropometric, physiological, and metabolic characteristics of normal weight and individuals with obesity (Ren et al., 2019). These studies showed that unlike serum Fet‐A, elevated serum pFet‐A concentrations were correlated to surrogate markers of insulin resistance, including serum insulin, HOMA‐IR, QUICKI, and adipose IR (Ren et al., 2019). However, to ease interpretation of data, demographic data (age, BMI, VO_2max_) are given in Table 1. In this study, we examined the effects of a single bout of moderate‐intensity exercise expending 500 kcals on serum Fet‐A, pFet‐A, and surrogate markers of insulin resistance/sensitivity. We show that blood glucose, serum insulin, HOMA, and QUICKI were not altered immediately after a single bout of exercise (Post Ex) in individuals who were normal weight or in individuals with obesity (Figure 2). Further, these surrogate markers were not altered 24 h Post Ex in individuals who were normal weight or with obesity. NEFA concentrations were significantly increased Post Ex in both normal‐weight participants and in individuals with obesity, and adipose IR increased Post Ex only in individuals with obesity. Further, this transient increase in NEFA and adipose IR returned to Pre Ex levels, 24‐h Post Ex. Serum Fet‐A concentrations were transiently increased Post Ex and returned to Pre Ex levels 24 h after the single bout of exercise in individuals with obesity, but not in individuals with normal weight. The phosphorylated form of serum Fet‐A, i.e., pFet‐A and the ratio of pFet‐A to total Fet‐A were not altered Post Ex or 24 h Post Ex compared with Pre Ex levels in individuals with obesity or normal weight (Figure 2). Independent of body weight (all participants), we observed a transient increase in serum Fet‐A concentrations, which was restored to Pre Ex levels 24 h after the single bout of exercise. However, serum pFet‐A and the ratio of pFet‐A to total Fet‐A were not altered Post Ex or 24 h Post Ex (Figure 2).

**TABLE 1:** Descriptive characteristics for normal weight (*n* = 11) and obese (*n* = 31) individuals

Variables	Total (*n* = 42)	Normal weight (*n* = 11)	Obesity (*n* = 31)	*p* value
	Mean ± SD	Mean ± SD	Mean ± SD	
Age (years)	43.3 ± 9.5	43.3 ± 10.7	43.3 ± 9.2	0.944
Height (cm)	177.1 ± 7.3	174.8 ± 6.3	177.8 ± 7.4	0.181
Weight (kg)	103.3 ± 26.1	72.6 ± 6.3	114.2 ± 12.2	<0.001
BMI (kg/m^2^)	32.8 ± 7.6	23.9 ± 2.0	36.0 ± 6.1	<0.001
Rest SBP (mm Hg)	124.5 ± 13.4	114.9 ± 11.1	127.9 ± 12.6	0.004
Rest DBP (mm Hg)	80.6 ± 9.2	75.1 ± 6.3	82.5 ± 9.3	0.019
VO_2max_ (ml/kg/min)	30.1 ± 7.1	36.0 ± 6.2	28.0 ± 6.1	0.001
VO_2max_ absolute (L/min)	2.97 ± 0.54	2.59 ± 0.39	3.10 ± 0.53	0.006

Note

*p* value shown for comparison of individuals with normal weight vs individuals with obesity.

Abbreviations: BMI, body mass index; DBP, diastolic blood pressure; SBP, systolic blood pressure; VO_2max_, maximum rate of oxygen consumption.

### 3.2 Serum glucose, insulin, Fet‐A, and pFet‐A responses to an oral glucose tolerance test

To further elucidate insulin sensitivity measurements, we compared glucose, insulin, Fet‐A, and pFet‐A responses to an OGTT conducted at both Pre Ex and 24 h Post Ex. Independent of body weight (all participants), glucose_AUC_, insulin_AUC_, Fet‐A_AUC_ (*p* = 0.0508), pFet‐A_AUC_, 2 h pFet‐A, and 2 h pFet‐A/Fet‐A were significantly decreased 24 h after a single bout of endurance exercise (Table 2). In individuals with obesity, we demonstrate an improvement in 24 h Post Ex glucose_AUC_, insulin_AUC_, insulin resistance index, Fet‐A_AUC_, and pFet‐A_AUC_, but not pFet‐A_AUC_/Fet‐A_AUC_ compared with Pre Ex (*p* < 0.05) (Table 2). Similarly, 2 h glucose, 2 h insulin, and 2 h pFet‐A, and 2 h pFet‐A/Fet‐A, but not serum Fet‐A, were significantly lowered 24 h after a single bout of exercise in individuals with obesity. In normal‐weight individuals, no significant changes were observed in response to a 24 h Post Ex OGTT in serum Fet‐A, pFet‐A, pFet‐A/Fet‐A, or insulin sensitivity measures compared with Pre Ex OGTT (Table 2).

**TABLE 2:** Metabolic indices, following an oral glucose tolerance test, in all participants (*n* = 42), individuals with normal weight (*n* = 11), and obesity (*n* = 31), before exercise (Pre Ex) and 24 h after a single‐bout of exercise (24  h Post Ex), expending 500 kcal

Variables	All participants (*n* = 42)		Normal weight (*n* = 11)		Obesity (*n* = 31)	
	Pre Ex	24 h Post Ex	Pre Ex	24 h Post Ex	Pre Ex	24 h Post Ex
Glucose_AUC_	16738 ± 3848	15396 ± 3343***	13947 ± 1802^a^	13154 ± 1542^a^	17718 ± 3849^b^	16219 ± 3463^c^
Insulin_AUC_	13901 ± 7528	12065 ± 6976***	5766 ± 3825^a^	4448 ± 3011^a^	16866 ± 6126^b^	14859 ± 5823^c^
NEFA_AUC_	41.6 ± 16.9	41.3 ± 17.9	23.4 ± 7.9^a^	25.3 ± 7.2^a^	48.3 ± 14.1^b^	47.2 ± 17.0^b^
Insulin resistance index	246.5 ± 158.2	194.8 ± 131.1***	80.4 ± 50.7^a^	59.2 ± 42.1^a^	306.1 ± 136.8^b^	244.6 ± 116.5^c^
Fet‐A_AUC_	38933 ± 7168	37924 ± 6644^#^	35110 ± 5436^a^	35614 ± 5484^a^	40212 ± 7197^b^	38772 ± 6910^a^
pFet‐A_AUC_	262.2 ± 147	227.9 ± 138*	170.6 ± 57.9^a^	185.9 ± 79.4^a^	296.3 ± 153.7^b^	243.3 ± 152.2^a^
pFet‐A_AUC_/Fet‐A_AUC_	1.41 ± 0.81	1.30 ± 0.89	1.00 ± 0.30^a^	1.07 ± 0.37^a^	1.57 ± 0.88^a^	1.38 ± 1.01^a^
2 h glucose (mg/dl)	107.2 ± 30.1	98.9 ± 23.7*	95.7 ± 22.5^a^	92.7 ± 13.2^a^	111.3 ± 31.4^b^	101.2 ± 26.5^a^
2 h insulin (μU/ml)	92.3 ± 64.9	73.6 ± 60.0**	40.1 ± 28.9^a^	33.7 ± 29.0^a^	109.8 ± 63.7^b^	88.6 ± 62.0^c^
2 h NEFA (mEq/L)	0.25 ± 0.21	0.21 ± 0.12	0.11 ± 0.04^a^	0.13 ± 0.08^a^	0.31 ± 0.21^b^	0.24 ± 0.13^b^
2 h Fet‐A (μg/ml)	325.2 ± 65.4	319.7 ± 64.5	293.9 ± 46.2^a^	295.4 ± 46.2^a^	335.6 ± 67.4^a^	328.7 ± 68.5^a^
2 h pFet‐A (scan units)	2.27 ± 1.30	1.61 ± 1.26**	1.50 ± 0.44^a^	1.53 ± 1.04^a^	2.51 ± 1.39^b^	1.65 ± 1.35^a^
2 h pFet‐A/Fet‐A	1.46 ± 0.89	1.09 ± 0.99**	1.06 ± 0.33^a^	1.01 ± 0.56^a^	1.60 ± 0.9^b^	1.13 ± 1.12^a^

Note

Data are shown as Means + SD. For comparisons between individuals with normal weight vs obesity, different letters in superscript following values indicate statistical significance either within the group or between groups, *p* < 0.05.

Abbreviation: AUC, area under the curve.

For comparison of Pre Ex vs 24  h Post variables among all participants, statistical significance is indicated as follows:

* *p* < 0.05,

** *p* < 0.01,

*** *p* < 0.001;

# *p* = 0.0508.

### 3.3 Correlation analysis

**FIGURE 3:**
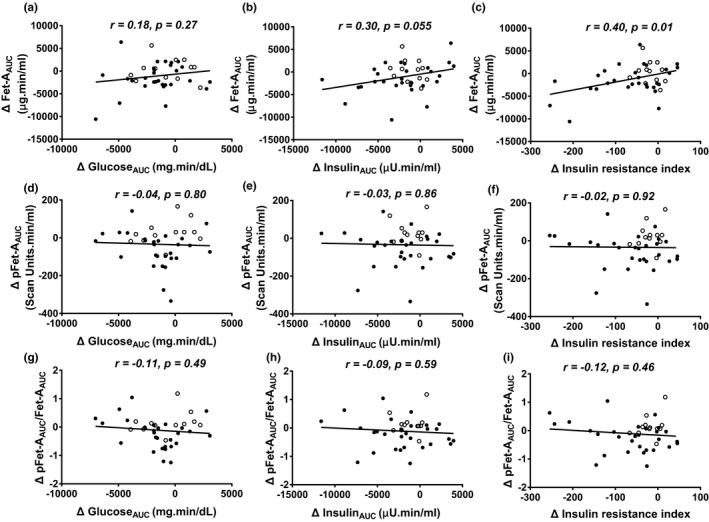
Area under the curve (AUC) for glucose, insulin, Fet‐A and pFet‐A was calculated following an oral glucose tolerance test to compare changes (Δ) from Pre Ex to 24h Post Ex in serum Fet‐A_AUC_ with Δ in glucose_AUC_ (a), insulin_AUC_ (b), and insulin resistance index (insulin_AUC_ × glucose_AUC_/10^6^) (c); Δ in pFetA_AUC_ with Δ in glucose_AUC_ (d); insulin_AUC_ (e); insulin resistance index (f); and Δ in pFet‐A_AUC_/Fet‐A_AUC_ with Δ in glucose_AUC_ (g); insulin_AUC_ (h); insulin resistance index (i) in all participants. Correlation was determined using Pearson product‐moment correlation coefficient (individuals with normal weight shown using open circles and individuals with obesity shown using closed circles)

We analyzed changes in 24 h Post Ex compared with Pre Ex concentrations and observed that changes in insulin resistance index were correlated with changes in serum Fet‐A_AUC_ (*r* = 0.40, *p* = 0.01) and changes in insulin_AUC_ tended to correlate with changes in serum Fet‐A_AUC_ (*r* = 0.30, *p* = 0.055), but not with serum pFet‐A_AUC_ or pFet‐A_AUC_/Fet‐A_AUC_ ratio (Figure 3). Further, fasting serum Fet‐A, pFet‐A, or pFet‐A/Fet‐A ratio were not correlated to changes in surrogate markers of insulin resistance/sensitivity comparing Pre Ex with Post Ex or Pre Ex with 24 h Post Ex (data not shown).

## 4 DISCUSSION

In this study, we report for the first time changes in serum Fet‐A and pFet‐A following a single bout of endurance exercise in both indivituals with normal‐weight and individuals with obesity. This study is unique, because it examines (a) alterations of serum Fet‐A, a negative acute‐phase reactant, immediately and 24 h after a single bout of exercise, (b) serum Fet‐A and pFet‐A responses independent of weight loss, and (c) association of serum Fet‐A and pFet‐A with the improvement of insulin sensitivty after a single bout of exercise.

Our studies indicate that, independent of body weight, a single bout of endurance exercise, expending 500 kcal, led to a significant transient increase in serum Fet‐A but not serum pFet‐A or pFet‐A/Fet‐A. These transient changes in serum Fet‐A were restored to normal levels 24 h after the single bout of exercise. Our studies showed that the ratio of serum pFet‐A/Fet‐A was not altered Post Ex or 24 h Post Ex in individuals with normal weight or obesity , suggesting that the dynamic responses observed may be attributed to changes in total serum Fet‐A and not its phosphorylated (pFet‐A) form. However, the transient increase in serum Fet‐A was not associated with the transient increase in NEFA concentrations. Light and moderate‐intensity exercise has been shown to increase delivery of NEFA in plasma, primarily from adipose tissue, and from intramyocellular triacylglycerol (Frayn, 2010). As liver has been identified as the key source for circulating Fet‐A, adipocytes have also been shown to secrete Fet‐A (Jialal & Pahwa, 2019). Thus, although it is possible that Fet‐A may be secreted by the adipocytes, in response to a single bout of exercise via muscle contraction, cytokine‐ or hormone‐mediated mechanisms (Khadir et al., 2018; Woltje et al., 2006), the relative contribution of these two tissue sources to circulation may be vastly different, because Fet‐A (AHSG) mRNA expression is at least 400 times higher in liver than in adipose tissue, based on consensus data from normalized expression levels for 55 tissue types created from three transcriptomics datasets (HPA, GTEx, and FANTOM5) (Uhlen et al.,).

Transient changes, caused by acute exercise, alters various aspects of muscle, adipose, and liver tissue function independent of body weight loss, and exerts beneficial consequences in metabolism and improves insulin sensitivity (Egan & Zierath, 2013; Hawley et al., 2014). Previous studies also demonstrated that a single bout of exercise has prolonged effects (up to 18 h) on postprandial response to food intake, such as reducing hepatic secretion of very‐low‐density lipoprotein, increasing triacylglycerol clearance, and increasing postprandial leg blood flow and glucose uptake (Gill et al., 2001a, 2001b; Malkova et al., 2000). The prolonged effects of single bout of exercise also have been found to alter trafficking of dietary fat and fat oxidation (Gill, Frayn, et al., 2001; Votruba et al., 2002). Our findings of improved insulin sensitivity 24 h after a single bout of moderate intensity exercise (60%–70% VO_2max_ and expending 500 kcals), as demonstrated through improvements in 2 h glucose, 2 h insulin, glucose_AUC_, insulin_AUC_, and insulin resistance index is consistent with previous reports (Hayashi et al., 2005; Hoene et al., 2009). Interestingly, our data shows that a single bout of exercise improves Fet‐A_AUC_, pFet‐A_AUC_, 2 h pFet‐A, 2 h pFet‐A/Fet‐A, in concert with improvements in 2 h glucose, glucose_AUC_ in individuals with obesity, but not in individuals with normal weight.

Recent studies by Malin et al., (2013) have shown that a 7‐day short‐term exercise training decreased fetuin‐A by 11% in adults with obesity, without a change in body weight or aerobic capacity. Similarly, our studies show significant reduction of Fet‐A_AUC_ and pFet‐A_AUC_ 24 h Post Ex, independent of body weight changes. Duncan et al demonstrated that 6 months of exercise improved insulin sensitivity and several markers of lipid metabolism in sedentary men and women, without a corresponding change in BMI, waist circumference, or cardiorespiratory fitness (Duncan et al., 2003). This suggests that alterations in fetuin‐A, independent of body weight changes, may play a role in insulin sensitivity.

We evaluated the sustained effects following a single bout of exercise and show improvements in 24 h Post Ex glucose_AUC_, insulin_AUC_, insulin resistance index, Fet‐A_AUC_, and pFet‐A_AUC,_ compared with Pre Ex, independent of body weight. Further, reductions in 24 h Post Ex Fet‐A_AUC_ were associated with a reduction in insulin resistance index. Post exercise improvements in insulin sensitivity and glucose tolerance may be explained by insulin‐dependent and insulin‐independent glucose uptake pathways, other serum factors, and autocrine/paracrine mechanisms (Goodyear & Kahn, 1998; Jessen & Goodyear, 2005). Insulin‐dependent GLUT4 translocation is the primary mechanism for increasing glucose tolerance during acute exercise (Kennedy et al., 1999; Wallberg‐Henriksson & Holloszy, 1984). Fet‐A, specifically pFet‐A, inhibits insulin signaling, insulin‐stimulated GLUT4 translocation, glucose uptake, and glycogen synthesis (Mathews et al., 2000; Ren et al., 2019). The reduction in serum Fet‐A_AUC_ and pFet‐A_AUC_ 24 h after the single bout of exercise suggests that it may contribute to decreasing inhibitory effects on insulin receptor phosphorylation and enhance downstream insulin signaling pathway, GLUT4 translocation, and increase glucose uptake. Acute exercise has also been shown to activate AMPK, which plays an important role in regulating glucose tolerance and energy metabolism (Kjobsted et al., 2017). However, additional studies are needed to characterize the effects of AMPK activation on regulation of Fet‐A and pFet‐A.

Although this study reports novel findings on alterations in circulating Fet‐A and pFet‐A following a single bout of exercise and its impact on markers of insulin sensitivity, these observations are limited to men. Additionally, this study was carried out in 11 individuals of normal weight and 31 individuals who were obese. Further, the sustained effect of exercise on Fet‐A, pFet‐A, and insulin sensitivity was evaluated immediately after and 24 h after the single bout of exercise. Another limitation is that, in this study, we used the oral glucose tolerance test to calculate the area under the curve for glucose and insulin, and not other measures of insulin sensitivity such as the euglycemic–hyperinsulinemic clamp study or the frequently sampled intravenous glucose tolerance test (Coates et al., 1995). Other studies have shown an improvement of insulin sensitivity persisting for a period ranging from 2 h (Mikines et al., 1988b), 4–6 h (Wojtaszewski et al., 2000), 12–16‐h (Devlin et al., 1987; Devlin & Horton, 1985; Heath et al., 1983), and up to 48‐h post exercise (Mikines, Sonne, et al., 1988; Perseghin et al., 1996). In our study, we did not refeed the 500‐kcal energy deficit from the single bout of exercise. Thus, there is a possibility that the observed alterations in Fet‐A and pFet‐A may at least be partially due to the energy deficit. Also, it will be of significant interest to examine the relationship of Fet‐A or pFet‐A and inflammatory cytokines, including IL‐6, IL‐1β, and TNF‐α following a single bout of exercise. Further, serum pFet‐A concentrations were assayed using a semiquantitative densitometric data from Western blots, which may be a limiting factor. Furthermore, Fet‐A is phosphorylated on both Ser120 and Ser312. As our antibody is specific to Ser312, we are unable to assess Ser120 phosphorylation, or its significance.

In conclusion, we demonstrate that, in individuals with obesity, serum Fet‐A was transiently elevated immediately after a single bout of exercise and restored to Pre Ex levels 24 h after a single bout of exercise. Furthermore, serum Fet‐A_AUC_, pFet‐A_AUC,_ glucose_AUC_, insulin_AUC_, and insulin resistance index were significantly decreased 24 h after a single bout of exercise compared with Pre Ex levels in individuals with obesity. Additionally, the reduction in 24 h Post Ex insulin resistance index was correlated with a reduction in serum Fet‐A_AUC_. Taken together, this suggests that alterations in serum Fet‐A and its phosphorylated form, Ser‐312 pFet‐A, may potentially contribute to the observed improvement in insulin sensitivity following a single bout of endurance exercise.

## CONFLICT OF INTEREST

The authors declared no conflicts of interest.

## AUTHOR CONTRIBUTIONS

PWJ and STM designed the study. GR, RB, TK, and AJM conducted the study. GR and STM drafted the manuscript, and GR, PWG, and STM analyzed the data and helped with interpretation of data, edited the manuscript, and had primary responsibility of final content. All authors read and approved the final manuscript.
